# Ridge number in bat ears is related to both guild membership and ear length

**DOI:** 10.1371/journal.pone.0200255

**Published:** 2018-07-25

**Authors:** Brian W. Keeley, Annika T. H. Keeley, Padraig Houlahan

**Affiliations:** 1 School of Forestry, Northern Arizona University, Flagstaff, Arizona, United States of America; 2 Department of Environmental Science, Policy, and Planning, University of California, Berkeley, Berkeley, California, United States of America; 3 Department of Physics, Embry-Riddle Aeronautical University, Prescott, Arizona, United States of America; 4 Coconino Community College, Flagstaff, Arizona, United States of America; University of Western Ontario, CANADA

## Abstract

The ears of many mammals have a set of uniformly spaced horizontal ridges that form groove arrays. Contact of coherent waves (e.g. acoustic waves) with a series of slits or grooves causes diffraction, which produces constructive and destructive interference patterns. Increases in signal strength will occur but will depend on the frequencies involved, the groove number and their separations. Diffraction effects can happen for a wide range of frequencies and wavelengths, but no array can diffract wavelengths greater than twice the groove separation, and it is for those wavelengths comparable in size with the groove separation that the effects are greatest. For example, when ridges in bat ears are 1 mm apart, the strongest influence will occur for a 1 mm wavelength which corresponds to a frequency of 343 kHz. If bats could use these wavelengths, it would help them to resolve objects or surface textures of about 0.5 mm. Given how critical acoustics are for bat function, we asked whether bats may be taking advantage of diffraction effects generated by the grooves. We hypothesize that groove number varies with bat foraging strategy. Examining 120 species, we found that groove number is related to both guild and ear length. Bats in guilds that glean prey items from foliage or ground have on average more grooves than bats in other guilds. Harmonics generated by echolocation calls are the most likely source for the wavelengths that would correspond to the groove separations. We apply the physical principles of wave reflection, diffraction, and superposition to support the hypothesis that acoustic responses generated from grooves may be useful to bats. We offer an explanation why some bat species do not have grooves. We also discuss the presence of groove arrays in non-echolocating Chiropterans, and five additional mammalian orders.

## Introduction

**Fig 1:**
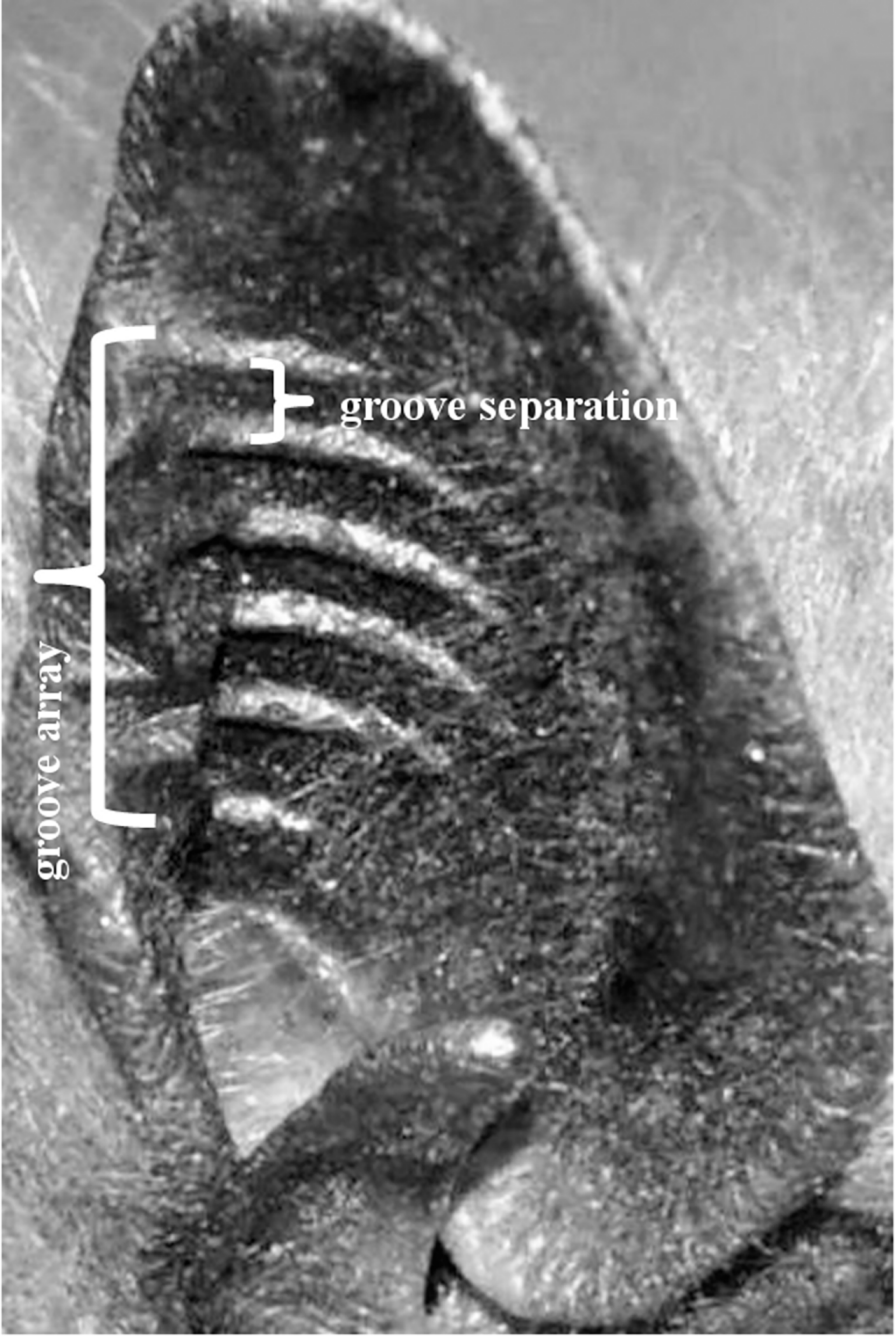
Bat ear with grooves. These six evenly spaced ridges in the ear of a big brown bats (*Eptesicus fuscus*) form an array containing five grooves.

Many nocturnal mammals rely on auditory cues for orientation, resource detection, and predator avoidance [1–4]. The presence of any morphological structure preceding the entry of sound waves into an organism’s auditory sensory system may produce useful acoustic information. The external ears of many species contain evenly spaced parallel ridges whose role is not clearly understood (Fig 1). Due to the specialized auditory system of bats, it has been suggested that the groove array may aid in hearing [5–10], but only two papers offer an explanation for an acoustic role associated with the grooves [11–12]. Here we study if species differences in groove numbers can be explained by different echolocation and foraging strategies. We discuss potential acoustic and mechanical roles of groove arrays.

**Fig 2:**
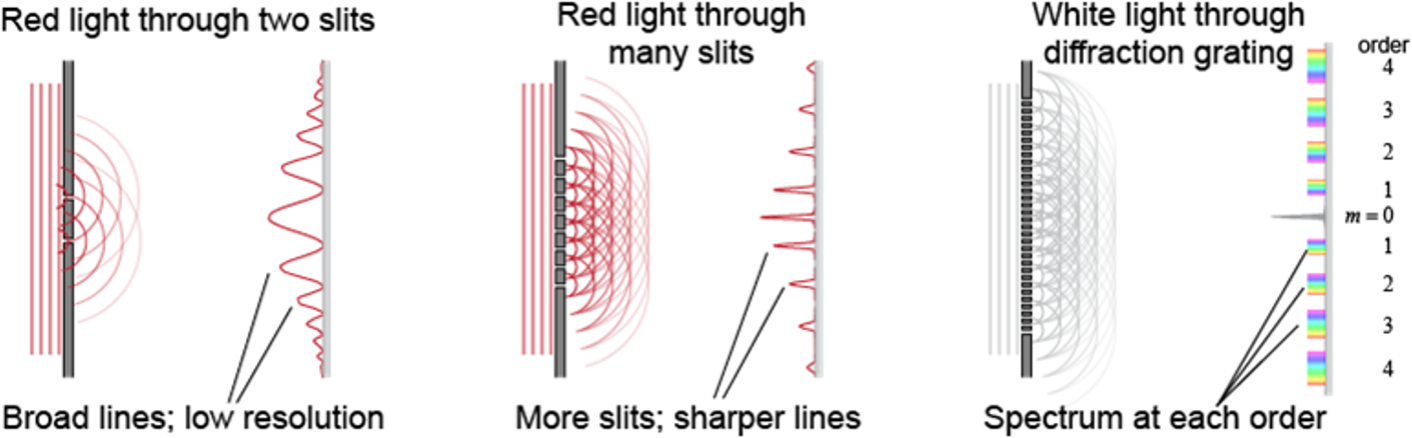
Diffraction effects from optical transmission gratings occur when light waves pass through slits. Each slit produces wavelets that create interference patterns which become more distinct as increasing groove numbers contribute to the effects. Multi-frequency waves such as depicted here with white light are broken into separate frequencies because the different wavelengths emerge at different angles. The same effect would occur with an acoustic frequency modulated (FM) sweep in bat ears deflecting from grooves (image courtesy of Pasco Scientific).

**Fig 3:**
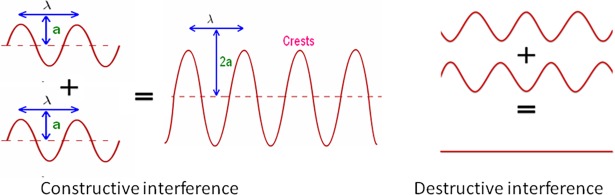
Constructive and destructive interference effects occur when two waves meet. Constructive interference will retain the frequency and double the amplitude when two identical waves of the same period and amplitude that are completely in phase meet. Destructive interference occurs when identical waves meet that are completely out of phase because it cancels the signal frequency and the amplitude to zero.

Slit or groove arrays are known to produce interference and diffraction effects when coherent waves pass through or reflect from them (Fig 2)[13]. The array produces a scattering pattern that varies with angle and depends on its properties: the number of slits or grooves, and the groove separation measured as the distance between two adjacent ridges, and the overall dimensions of the array (Fig 1). For example, a single groove does not cast a simple acoustic shadow but will bend and distort the path of reflected waves beyond their specular reflection angle to create peaks (Fig 2) where constructive and destructive interference happens (Fig 3). With the addition of more grooves, the peaks become more pronounced and narrower. The center peak is the most pronounced and the height of the other peaks decreases with increasing angular separation from the axis of symmetry (Fig 2). The intensity of the peaks is a function of the number of slits or grooves and angular separation from the center (Eq 1).

$$I=4I_{0}{\left[\frac{sin(\pi \frac{a}{\lambda }\sin\;\theta )}{\frac{\pi a}{\lambda }\sin\;\theta }\right]}^{2}{\left[\sum_{p=1}^{n/2}cos\;(\left(2p-1\right)\pi \frac{d}{\lambda }\sin\;\theta )\right]}^{2}$$ (1)

where *I* is the peak intensity, I_0_ is the incident intensity, *a* is the slit or groove width, λ is the wavelength, and θ is the outgoing angle, *p* is a parameter to count grooves, *n* is the number of slits or grooves, and *d* is the distance between the ridges [13]. Eq (1) includes a product of 2 squared terms. Since it is well known that SINC(x) = SIN(x)/x = 1 when x is zero, and the first is the square of a SINC() function, so when θ = 0, the [SINC()]^2^ term is 1. And when θ = 0, all the COS() terms in the second term of the equation are unity, and the summation reduces to n/2, therefore at the central peak (θ = 0) Eq (1) reduces to (Eq 2)

I=4I0(n2)2=I0n2 (2)

Therefore, comparing two gratings where we double the number of grooves, one with 4 and another with 8 grooves, the respective peak intensities would be 16 I_*0*_ and 64 I_*0*_ so the intensity has changed as the square of the number of grooves.

Therefore, an array with 12 grooves produces a very narrow central peak with the intensity 144 times greater than that from a single groove. Note also that diffraction effects are more pronounced the more monochromatic (single frequency) the source. While for a given grating diffraction effects can happen for a wide range of frequencies and wavelengths, no grating can diffract wavelengths greater than twice the groove separation, therefore it is for those wavelengths comparable in size with slit separation that the effects are greatest. According to the relationship *f = c/* λ where *f* is the frequency, and *c* is the speed of sound (343 m/s), a 1 mm wavelength (λ) will correspond to a frequency of 343 kHz. A 1mm diffraction grating will begin to cause diffraction effects at ~172 kHz and above with the peak response occurring at 343 kHz.

It is important to appreciate that lobes produced by plane waves impacting flat diffracting gratings are described as lying in a plane orthogonal to the grating surface and parallel to the grooves. Diffraction effects redirect wave energy transversely from the grooves in the arrays. Because the array is oriented horizontal to the ear’s long axis and centered above the ear canal, the diffraction lobes are directed toward the ear drum. Note that when an array is present in an ear, the grooves are always oriented in this direction. If the grooves in an array were vertical, the grooves would direct most of the energy away from the ear canal and would be unable to provide benefit.

Given how critical acoustics are to bats, it is possible that bats are taking advantage of the effects generated by the grooves. While the overall shape of the ear focuses all sound frequencies in a conventional way, we postulate that the grooves are concentrating diffracted frequencies into primary diffraction lobes aligned with the reflection angle, which is controlled by groove dimensions and number.

To take advantage of the diffraction effects created by 1mm grooves in some way, bats would need to be able to make use of frequencies greater than 172 kHz with a peak response occurring at 343 kHz. Although it would be surprising to learn that bat species could hear frequencies as high as 343 kHz, there is evidence that the hearing of some species goes as high as 250 kHz. For example, Myotis oxygnathus responded to sounds as high as 250 kHz, which was the highest frequency the equipment was able to test [14]. Some bat species (e.g. Kerivoulinae ssp. and Murininae ssp. in the family Vespertilionidae) have also been documented to emit calls with fundamental frequencies (as opposed to harmonics which they also emit) up to 292 kHz [15]. We suggest that the bats should be able to hear most or all of the fundamental frequencies they emit but they may not be able to hear portions of the harmonics without some form of assistance. Microphones and sampling and processing rates used in most studies were limited to detect frequencies up to but not above 250 kHz [16–20], and even if the equipment were capable to accurately sample at those rates, the microphone would need to be close enough to the source to pick up all frequencies evaluated up to 343 kHz (or above) to overcome attenuation levels. If the bats can process information from such high frequencies, it should contribute to their ability to locate objects with high precision [6–7] and to discern very fine structural detail [21].

Target resolution is related to wavelength [21]. Wavelengths up to 100 kHz will only provide information about the size of a small prey item ~1.5mm in size. However, it may be important for bats to know greater detail about a prey item than just its size. Many bats emitting calls with fundamental frequencies well below 100 kHz are able to perform complicated tasks in complex structured environments, suggesting they have access to greater detail than the fundamental frequencies can provide. Studies on target resolution have concluded that *Glossophaga soricina* can discriminate structural differences as small as 380 μ m using calls with three overlapping harmonics that starts with a fundamental FM sweep of ~55–95 kHz. The authors conclude that the most likely source of that ability comes from wave interference patterns generated from suitable sized wavelengths of ~190 kHz found in the third harmonic [21]. If harmonics are the source of the wavelengths necessary for *G*. *soricina* to discriminate that level of detail, then it means the intensities within harmonics will be greatly diminished and that the bat is using those frequencies somehow. The diffraction effects attributable to the number of grooves (*G*. *soricina* has 6 grooves) within the arrays may be making it possible to increase the intensities to usable levels. But that still would not explain how many species would be able to use frequencies generated as harmonics that could be well above their hearing range. We could not find information on the hearing range for *G*. *soricina*. Using the principles of wave behavior, we offer a possible explanation of how the grooves may be assisting the bats to access information from frequencies that may be above their hearing range.

Echolocating bats have evolved highly specialized auditory structures including variability in ears along with a diversity of feeding strategies. In this study, we evaluate if the groove number varies with bat foraging strategies. We hypothesize that the grooves reflect incoming acoustic waves in patterns that make them useful to bats and discuss the wave behaviors that may play a role. Throughout this manuscript we consider a 1mm separation array for the ease of maintaining a consistent discussion, but it must be kept in mind that groove separation in bats can be above and below this value. We also discuss the presence of the groove arrays in the ears of non-echolocating bats, and four additional orders of mammals.

## Methods

To determine the number of grooves in the ears of bats, we took photos of bats that we captured in Arizona, United States, and Nicaragua. Animals were captured and handled following guidelines of the American Society of Mammalogists [22] and with approval of Northern Arizona University (NAU) Institutional Animal Care and Use Committee. Nicaraguan bats were captured and handled under Nicaraguan Permit Autorización No. 015–122011.

We measured groove separation in bat ears by laying a ruler next to the grooves when taking photographs. From these photos, we counted the grooves and measured groove separation as the distance from ridge peak to peak (Fig 1). We counted a groove when it was part of a series that forms a groove array within an ear. We also counted the number of grooves from photographs posted on the Internet if we had sufficient information available to assign the species to a guild. To qualify for a count, the grooves in the ear had to be clearly visible in the photo. Within a species, we found that the number of visible grooves can vary by up to three grooves and/or include partial and shortened grooves which may occur from natural variation, from the inability to see all grooves in a photograph or while held in hand due to unfavorable light conditions. We were not always able to view multiple photographs of sufficient quality to cross-check all species, so instead of trying to use an average groove number, we chose the highest number of grooves we encountered, but acknowledge that differences do exist. We measured ear length of bats we captured; for bats we did not capture, we looked up the average ear length in the literature. We assigned 120 bat species with and without grooves to guilds following the classification by Denzinger & Schnitzler [23], who defined seven guilds based on habitat type, foraging mode, and echolocation behavior (Table 1).

**Table 1:** Bat guild description.

	Guild	Description
1	open space, aerial	• hunt for air borne prey in open space
2	edge space, aerial	• hunt for air borne prey in edge space; • find food in the vicinity of background targets
3	edge space, trawling	• hunt for insects drifting on or flying just above calm water or hunt for fish
4	narrow space, flutter detecting	• search for prey in narrow space; • detect echoes of the beating wings of insects
5	narrow space, passive gleaning	• search for prey (arthropods, small vertebrates) in narrow space; • rely on prey generated sounds to localize the prey; • vision may also play a role in prey detection
6	narrow space, active/passive gleaning	• frugivorous and nectarivorous bats • use odor for rough localization of the • food source and echolocation for precise localization
7	narrow space, active gleaning	• use echolocation to find prey (insects) on or near background objects so far only one bat species has been identified to use this tactic (*Micronycteris microtis*)

(adapted from Denzinger & Schnitzler)[22].

We computed Pearson's correlation coefficients to determine the relationship between ear length and groove number within the pooled data set and within each guild separately. We analyzed the effect of guild and ear length on groove number using generalized linear models in R [24]. We only included guilds 1–6 in the analysis because guild 7 currently only contains one species [23]. A priori we built three candidate models: the number of grooves is related to either the guild, or ear length, or both. We modeled the Poisson distribution with the natural logarithm as the link function in the R base package. We selected the best model based on the lowest AICc ranking corrected for small sample size (AICc) [25]. We conducted log-likelihood ratio tests to test whether the smaller model is the ‘true’ model. This test compares the log likelihoods of two models; if the difference is statistically significant then the model with more variables fits the data significantly better than the model with fewer variables. We applied a Mann-Whitney U test to compare groove number among six feeding guilds. We adjusted the p-value for multiple comparisons using the Bonferroni correction. To determine the presence or absence of groove fields in the ears of a diversity of other mammals, we reviewed photographs on the internet.

## Results

The number of grooves in bat ears varied between 0 and 20 among the 120 bat species that we assigned to guilds (Table 2). Groove separation measurements for the bat species that we handled were 0.4–1.3 mm (Table 2).

**Table 2:** Guild, number of grooves, average groove separation, and ear length of 120 bat species included in the study.

Guild	Species	Groove separation	Groove number	Ear length (mm)
1. open space,	Eptesicus andinus*		5	14.8
aerial	*Eumops glaucinus*^	0.6	4	20
	*Eumops perotis*^		5	41.5
	*Eumops underwoodi*^	0.6	6	28.5
	*Lasiurus cinereus*^	0.5	4	18
	*Molossus molossus*^	0.5	8	13
	*Molossus pretiosus*^	0.4	9	16.5
	*Molossus rufus*^		9	17.5
	*Molossus sinaloa*^	0.5	9	13.5
	Nyctalus noctula*		5	18
	*Nyctinomops femorosaccus*^		6	23
	*Nyctinomops laticaudatus*^		5	17.9
	*Nyctinomops macrotis*^	0.7	5	28.5
	Rhinopoma microphyllum*		9	21.6
	*Tadarida brasiliensis*^		5	15
	Tadarida teniotis*		9	26.4
2. edge space,	Eptesicus bottae*		4	18.5
aerial	*Eptesicus furinalis*^	0.5	3	13
	*Eptesicus fuscus*^	0.7	5	15
	Eptesicus serotinus*		5	17.5
	*Euderma maculatum*^	1.3	13	47.5
	*Lasiurus borealis*^		3	10.5
	*Lasiurus ega*^	0.7	6	18.5
	Myotis melanorhinus*		3	15.5
	Myotis mystacinus*		3	14.5
	Myotis nattereri*		3	20
	*Myotis occultus*^	1	5	13.3
	*Myotis riparius*^	1	6	12.5
	*Myotis thysanodes*^	0.9	17	18
	Myotis velifer*		4	16
	*Myotis volans*^	1	4	12.5
	*Parastrellus hesperus*^		4	11
	*Perimyotis subflavus*^		3	12
	Pipistrellus kuhlii*		4	14
	Pipistrellus pipistrellus*		3	12
	*Pteronotus personatus*^	0.5	5	14.1
	*Pteronotus gymnonotus*^	0.7	6	14.5
	*Rhogeessa tumida*^	1	3	12.5
	*Saccopteryx bilineata*^	0.5	12	15
	Saccopteryx leptura*		10	13.5
	Kerivoula krauensis*		0	12.1
	Kerivoula papillosa*		0	16.7
	Kerivoula pellucida*		0	15.4
	Kerivoula minuta*		0	10.7
	Phoniscus atrox*		0	11.1
	Murina suilla*		0	12.3
	Natalus stramineus*		0	13
	Fruriptera horrens*		0	11
3.edge space,	Macrophyllum macrophyllum*		7	12.5
trawling	Myotis albescens*		2	14.2
	Myotis capaccinii*		3	13.5
	Myotis dasycneme*		3	15.5
	Myotis daubentoni*		2	12.4
	Myotis emarginatus*		5	18.5
	Myotis vivesi*		7	20.4
	*Myotis yumanensis*^		4	12.8
	*Noctilio albiventris*^		7	23
	*Noctilio leporinus*^	1	7	21
4.narrow space,	*Pteronotus parnelli*^	0.7	2	23
flutter detecting	Rhinolophus blasii*		7	21
	Rhinolophus clivosus*		7	23.5
	Rhinolophus euryale*		7	22
	Rhinolophus ferrumequinum*		9	26
	Rhinolophus hipposideros*		6	17.5
	Rhinolophus mehelyi*		10	20.5
	Rhinolophus paradoxolophus*		8	33.9
5. narrow space,	*Antrozous pallidus*^	1	9	29
passive gleaning	*Chrotopterus auritus* *		20	44
	*Corynorhinus rafinesquii*^		9	32
	*Corynorhinus townsendii*^		10	34.5
	*Lophostoma brasiliense*^	0.6	14	23.5
	Lophostoma silvicolum*		16	34.5
	*Macrotus californicus*^		15	25
	Micronycteris hirsuta*		11	26
	Micronycteris megalotis*		10	23.3
	*Micronycteris minuta*^	0.6	13	21
	*Micronycteris schmidtorum*^	0.6	8	17
	Mimon bennettii*		15	37
	Mimon crenulatum*		6	25.5
	*Myotis auriculus*^	1	6	19
	Myotis bechsteinii*		7	24.5
	Myotis blythii*		8	26.5
	*Myotis evotis*^	1	7	15.5
	Myotis myotis*		6	28.5
	*Myotis septentrionalis*^		7	28.5
	Otonycteris hemprichi*		11	16.5
	*Phylloderma stenops*^	1	7	41
	*Phyllostomus elongatus*		10	30
	Plecotus alpinus*		19	31.5
	Plecotus auritus*		18	36
	Plecotus austriacus*		20	36
	Tonatia bidens*		15	32
	*Tonatia saurophila*^	0.8	13	23.5
	*Trachops cirrhosus*^		13	33
	Trinycteris nicefori*		7	18
	Vampyrum spectrum*		10	40.5
	Nycteris grandis*		0	31.5
6. narrow space,	*Artibeus literatus*^		8	18.5
passive/active gleaning	Artibeus obscurus*	0.4	10	20.4
	*Artibeus phaeotis*^	0.6	7	16
	Artibeus planirostrus*		8	20.5
	*Carollia brevicauda*^		5	18
	*Carollia perspicillata*^		8	19.5
	*Choeronycteris mexicana*^		8	16.5
	Dermanura phaeotis*		8	16
	*Dermanura watsoni*^	0.4	6	15.5
	*Glossophaga soricina*^		6	10.5
	Leptonycteris nivalis*		2	15
	Leptonycteris yerbabuenae*		5	18.5
	Mesophylla macconnelli*		6	13.5
	*Phyllostomus discolor*^	0.6	9	21
	*Phyllostomus hastatus*^	1	8	31
	*Platyrhinus helleri*^	0.5	8	17
	*Rhinophylla fischerae*		6	13
	Sturnira lillium*		5	16
	*Uroderma bilobatum*^	0.5	8	12.5
	*Vampyressa pusilla*^		6	13
	Vampyriscus brocki*		7	13.4
7. narrow space, active gleaning	Micronycteris microtis*	* *	11	19

*Groove number was determined using photos from the internet and mean ear length was determined from the literature

^ Groove number and ear length were determined from captured bats.

The Pearson’s correlation coefficient describing the relationship between ear length and groove number was 0.6 in the pooled data set. Within the guilds the coefficient ranged from -0.18 to 0.68 (Table 3).

**Table 3:** Groove number is related to ear length in some bat guilds.

Guild	Pearson correlation coefficient	p value
**1**	-0.18	0.74
**2**	0.50	<0.01*
**3**	0.68	0.02*
**4**	0.18	0.33
**5**	0.40	0.01*
**6**	0.39	0.04*
**overall**	0.60	<0.01*

*Low p-values, marked with an asterisk, correspond to a statistically significant relationship

**Fig 4:**
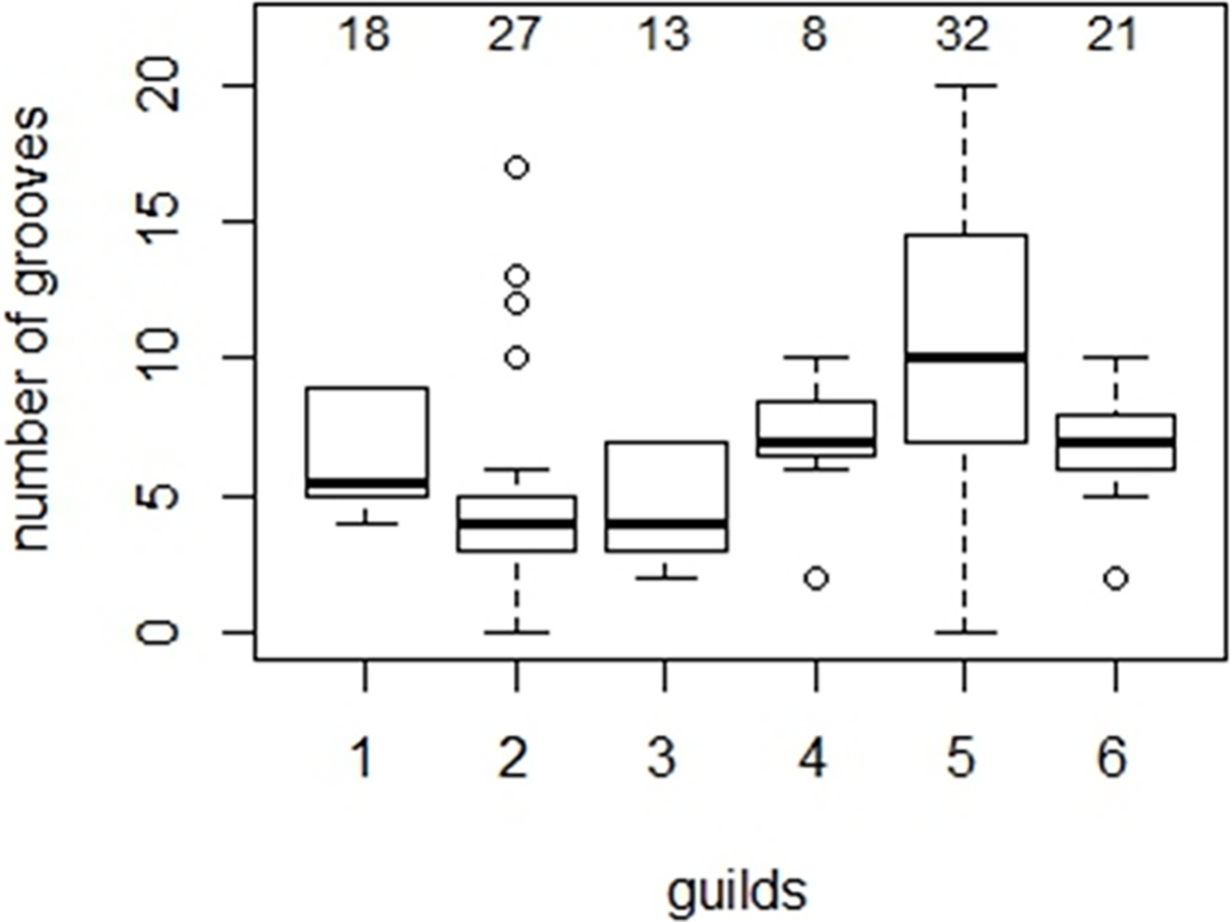
Distribution of groove number for species in each bat guild. 1—open space, aerial; 2—edge space, aerial; 3—edge space, trawling; 4—narrow space, flutter detecting; 5—narrow space, passive gleaning; 6—narrow space, active/passive gleaning. The sample size is given above the boxes for a total of 119 species within the six guilds. Guild 7 was not included in the analysis because it only contains one species.

The number of grooves modeled as guild and ear length is the best model, followed by the number of grooves modeled as ear length and number of grooves modeled as guild alone (Table 4). Log-likelihood ratio tests show that model 1 fits the data significantly better than model 2 and 3, respectively. Bats that search for prey in narrow spaces and take advantage of prey-generated sounds (guild 5) have a significantly greater mean number of grooves compared to the other guilds (Fig 4, Table 5).

**Table 4:** Results of AIC and log likelihood ratio tests to determine the model that best explains the number of grooves in bat ears.

Model	AICc	ΔAICc	Likelihood ratio test statistic	Degrees of Freedom	p
**#grooves ~ guild + ear length**	633.68	0			
**#grooves ~ ear length**	656.76	23.38	35.973	1	<0.001
**#grooves ~ guild**	658.88	25.17	23.320	1	<0.001

**Table 5:** Results of Mann-Whitney U tests for difference in groove number between all pair combinations from six guilds. See Table 1 for guild definitions and sample sizes.

	U	p
**Guild 1 ~ Guild 2**	416	0.02*
**Guild 1 ~ Guild 3**	103.5	1.00
**Guild 1 ~ Guild 4**	49	1.00
**Guild 1 ~ Guild 5**	83	<0.01*
**Guild 1 ~ Guild 6**	144	1.00
**Guild 2 ~ Guild 3**	122	1.00
**Guild 2 ~ Guild 4**	54	0.15
**Guild 2 ~ Guild 5**	111	<0.01*
**Guild 2 ~ Guild 6**	127	<0.01*
**Guild 3 ~ Guild 4**	18	1.00
**Guild 3 ~ Guild 5**	31	<0.01*
**Guild 3 ~ Guild 6**	44	0.32
**Guild 4 ~ Guild 5**	60	0.39
**Guild 4 ~ Guild 6**	92	1.00
**Guild 5 ~ Guild 6**	514.5	<0.01*

P-values were adjusted for multiple comparisons.

(*Low p-values indicate statistically significant differences.)

Our cursory review of mammals other than bats found that at least 12 other species of mammals in 4 orders also have recognizable groove fields associated with their ears (Table 6).

**Table 6:** Mammal species (other than bats) with grooves in their ears.

Order	Common name/Genus Species
**Primates**	gray mouse lemur *Microcebus murinus*
	weasel lemur *Lepilemur mustelinus*
	lesser bushbaby *Galago moholi*
**Dermoptera**	Phillipines flying lemur *Cynocephalus volans*
	Sundae flying lemur *Galeopterus variegatus*
**Rodentia**	Northern grasshopper mouse *Onychomys leucogaster*
	Gambian pouched rat *Cricetomys gambianus*
**Marsupiala**	common opossum *Didelphis marsupialis*
	mouse opossum *Mormosa robinson*
	Eastern barred bandicoot *Perameles gunii*
	wooley opossum *Caluromys philander*

## Discussion

Our data show that groove number is related to both guild and ear length. Bats in the narrow space, passive gleaning guild (guild 5) have on average more grooves and some of the longest ears compared to bats in the other guilds (Fig 3, Table 3). Bats in this guild often use prey-generated sounds to detect prey, and then glean prey items from the foliage or ground [23, 26–28]. In some cases, short, high frequency echolocation calls are used during the gleaning attack [28–29]. To capture the soft, prey-generated sounds gleaning bats have, in proportion to body size, large and wide pinnae [1, 3]. Consequently, ear size and number of grooves are confounded, which is reflected in the best model: groove number is a function of groove number and ear size. However, not all large ears have many grooves and not all short ears have few grooves. For example, Sinaloa mastiff bats (*Molossus sinaloa)* have relatively short 13 mm-pinnae with 9 grooves, whereas some serotine bats (*Eptesicus serotinus)* have long 20 mm-pinnae with only 5 grooves. If these differences in ear size and groove number are related to acoustic needs, it may lend support to the hypothesis that the groove number may have a stronger link to foraging strategy than ear length.

Diffraction gratings can work with a range of wavelengths, but no grating can diffract wavelengths greater than twice the groove separation. The general diffraction equation for incident waves of arbitrary angle is (Eq 3)

$$d\;(\sin({ϴ}_{in})-\sin({ϴ}_{out}))=m\lambda$$ (3)

where θ_in_ and θ_out_ are the incident and outgoing angles, d is the groove separation, and m (0, +/- 1, +/-2, +/- 3…) is the diffraction order. For normally incident waves, and first order diffraction, θ_in_ is 0 and sin(θ_out_) = θ /d. Because outgoing waves with θ_out_ greater than 90 degrees are not possible, sin() must be less than 1, and the maximum diffracted wavelength for normal incidence has to be less than the groove separation d. By allowing non-normal incident waves, the constraint can be relaxed to λ< 2d. Therefore, under normal incidence, waves longer than d will simply be reflected, but not diffracted. For extreme incidence, waves up to 2d can be diffracted. This implies that a 1mm based groove array should cause diffraction effects to begin for frequencies ~>172 kHz with the peak response occurring at 343 kHz. In addition, because different wavelengths emerge from the array at slightly different angles, bats would have the ability to make micro-adjustments to the array angle to monitor different frequencies being diffracted from the array.

In the bat species with grooves that we examined, groove separations varied between 0.4 and 1.3 mm (Table 2). Diffraction gratings with these separations would have their peak response intensify frequencies between 857 kHz and ~264 kHz, but because diffraction cannot occur for wavelengths greater than twice the groove separation, diffraction would begin occurring at half of these values. Many bat echolocation calls consist of fundamental frequencies and harmonics [2, 6, 30]. In many species, the fundamental frequencies (= first harmonic) of the echolocation calls are between 25–100 kHz [6]. Each harmonic is a multiple of the fundamental frequency. For example, if the first harmonic (fundamental) is 100 kHz the second and third harmonics are 200 and 300 kHz, respectively. Echolocation calls with up to seven harmonics have been documented [26]. Although frequencies of 343 kHz or above have not yet been documented, we expect bat vocalizations to contain the acoustic frequencies that match the groove separations or at a minimum cannot be greater than twice the size of the separation measurement which would start at ~172 kHz or greater for a 1mm based array. Such vocalization frequencies corresponding to groove separations are probably generated as harmonics. These frequencies would need to have enough intensity to return as an echo to contact the grooves despite attenuation. And if these frequencies do contact the array they are probably being diffracted.

**Fig 5:**
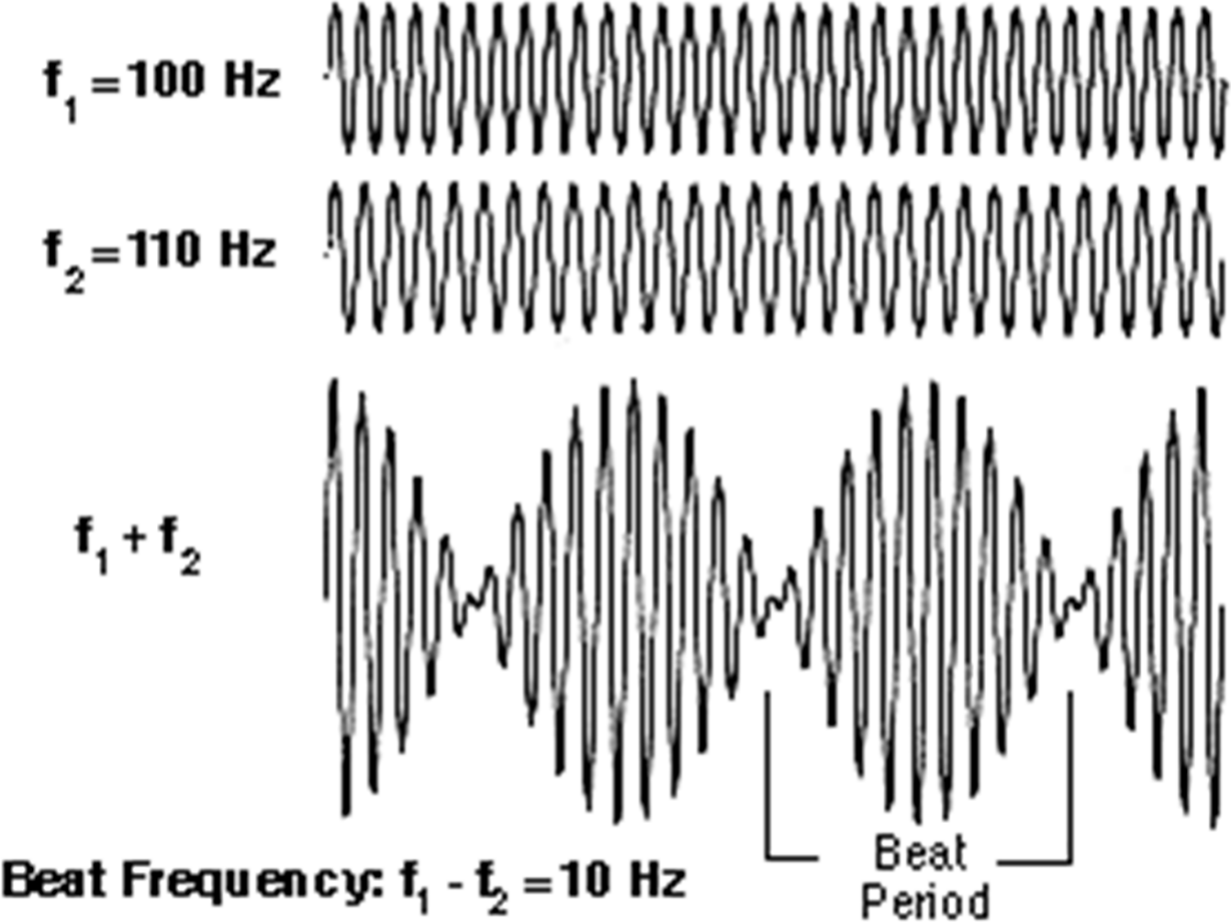
The phenomenon of sound waves of different frequencies creating a beat frequency is called superposition. For example, a frequency of 100Hz integrates with a frequency of 110Hz to form a 10Hz beat frequency [13]. Image courtesy of SFU School of Communication.

Although it appears that some species are capable of vocalizing and possibly hearing frequencies as high as ~290 kHz [15], it is unlikely that many species with 1mm sized grooves in their ears would have those abilities, especially for the ideal frequency of 343 kHz. There is also the possibility that the frequencies being intensified by diffraction are beyond the bats’ hearing range and do not provide any useful information to the animal. But it seems counterproductive for a group of animals that have invested so heavily in the evolution of acoustic abilities as bats that if acoustic information is being intensified by structures within their ears that there would not be some method of making use of such valuable information. Therefore, in an attempt to address the issue of how bats may be using frequencies that appear to be above their hearing range, we offer a very hypothetical approach that can be partially supported by the physical principles described for wave superposition. While the simplest hypothesis is that 1mm grooves are producing diffraction-based benefits for the bat at nominal frequencies of about 343 kHz, we also consider other possible scenarios that would create lower frequencies within the audible range through the phenomenon of beat frequency generation. Because beat frequencies require two different frequencies to interfere with each other, and the resulting beat frequency is simply the difference between them, which can be substantially smaller than either of the originals, beat frequencies may then be a mechanism for translating high frequency signals into lower (audible) frequency ranges. The closer the original frequencies, the lower the resulting beat frequency (Fig 5).

Although we describe a highly speculative scenario here, we suggest that, because of issues associated with attenuation for frequencies capable of being diffracted, they would probably only be detectable in the final moments before contact which is usually when many echolocating bats employ the feeding buzz. If lower frequency beats are created from returning echoes during this rapid series of closely linked, but constantly changing pulses in the feeding buzz, they may take part in what has been described as “stroboscopic sonar illumination”, as a bat approaches a target [31]. Linking these pulses together in a long series may provide a continually updated information stream that helps to monitor prey location, size, movements, and may help to discern fine structural details. The source for the specific frequencies producing the beats has yet to be determined but could result from diffracted frequencies within the sweep, from harmonic overlap, from differences generated by the Doppler Effect, or, since there is no requirement they be from the same pulse, possibly from outgoing emissions. We were able to find studies where penguins appear to be using beat frequencies created from a dual vocalization process as a means of creating lower frequency sounds that penetrate farther through crowded nesting colonies and also produce unique audible patterns that assist in recognizing individuals within the visually restricted conditions of the colony [32–33]. Hence, it does not seem unrealistic that wave superposition could provide a benefit to bats in addition to benefits from diffraction effects. The key element to this superposition concept is that the frequencies linked to the groove separations may be providing useful information at audible frequencies to the bats.

When measuring the groove separations, we noticed that the bats can change the shape of their ears, including the groove arrays, sometimes extending them or contracting them which altered the groove separation measurements. Schneider [34] found that the ridges contain innervated bands of muscles. This may indicate that bats have some ability to control the spacing and possibly the shape of the arrays. If bats can increase or decrease the groove separations, it may enable them to tune into specific wavelengths. A shift in separation of just 0.5 mm would considerably expand the bats’ ability to monitor a wider range of frequencies. In addition, the grooves appear to assist with supporting and folding the ear in many species [5]. This is especially noticeable for species with very large ears that have groove arrays spanning most of the ear (e.g. *Euderma maculatum*, *Corynorhinus*, *Plecotus sp*.). The ability to retract the ears during roosting may help to reduce injury, conserve energy, and reduce water loss [5]. However the grooves do not appear to always be necessary to provide support or assist the folding process as some species with large ears such as in the genus *Nycteris* have no grooves; conversely, other species such as *Artibeus gnomus* have many grooves but small ears that may not need to be folded. Therefore the grooves might have multiple purposes; assist the ear to flex during folding, and to maneuver the array in a manner that directs sound energy into the ear and to offer diffraction effects.

Some echolocating bat species have indistinct or no grooves in their ears, including species in the genera *Kerivoula*, *Murina*, *Ceolops*, *Natalus*, *Fruripterus*, *Thyroptera* and *Nycteris*. Bats without grooves have been shown to produce some of the highest fundamental frequencies documented in the vocalizations of all echolocating bats. For example, echolocation calls of bats in the genus *Kerivoula* have been recorded as high as 292 kHz [15] with bandwidths that span up to 155 kHz (Table 7). The use of smaller wavelengths has been attributed to the ability to discriminate directional and structural details [21]. A study of *Glossophaga soricina* attributed the ability to discern minute structural details to the use of ~190 kHz found in the third harmonic [21]. Presumably, an echolocating bat can either generate wavelengths that meet their needs directly as a fundamental or would have to access wavelengths generated within harmonics. But because harmonics typically have diminished intensities, a groove array may be necessary to intensify those weaker signals to usable levels. If bats require the level of detail that comes with the use of wavelengths at ~190 kHz, then a bat without grooves may need to compensate by increasing the fundamental frequencies in its vocalizations. Therefore bat species with groove-less ears and those with grooves may be using two different vocalization methods to accomplish similar tasks.

**Table 7:** Frequencies of echolocation calls of groove-less species.

Family	Species	Maximum and minimum frequencies of echolocation calls (kHz)	Bandwidth	Source
**Vespertilionidae**	*Kerivoula intermedia*	189 to 90	99	[17]
** **	*K*. *krauensis*	201 to 67	126.5	[17]
** **	*K*. *papillosa*	192 to 68	115.94	[17]
** **	*K*. *pellucida*	226 to 59	155.62	[17]
** **	*K*. *minuta*	175.2 to 85.8	89.4	[35]
** **	*Phoniscus atrox*	169 to 72	92.48	[17]
** **	*P*. *jagorii*	178.94 to 74.67	96.22	[17]
**(indistinct grooves)**	*Murina cyclotis*	178 to 57	115.41	[17]
** **	*M*. *suilla*	164 to 94.01	94.01	[17]
	*M*. *aenea*	152.4 to 43.3	109.1	[35]
**Hipposideridea**	*Coelops frithii*	194 to 113	81	[36]
**Nycteridae**	*Nycteris grandis*	114–17	97	[37]
**Natalidae**	*N*. *stramineus*	152.8 to 79.8	73	[38]
**Fruripteridae**	*Fruripterus horrens*	191.3 to 135.1 and 190.5 to 128.6	56.2 to 68.4	[39]
	*Amorphochilus schnablii*	163 to 87.3 and 151.4 to 67.2	75.7 and 84.2	[39]

Bats typically assigned to Guild 5 often glean prey from the ground or foliage. There are gleaning bats with some of the highest groove numbers for all animals we reviewed and a few species (possibly the entire family of bats: Nycteridae) appear to have no grooves. Information on the vocalizations for most of the grooveless species are not readily available but it appears that at least *Nycteris grandis* of the grooveless gleaning bats does not appear to use the distinct vocalization differences when compared to grooved species like those we just described for aerial foraging strategies (Table 5). In order to compare gleaning bats that have grooved ears to those that do not, we need to know if prey-generated sounds contain suitable wavelengths that are able to be diffracted. In separate studies on prey-generated sounds, relatively large arthropods (eg. beetles and scorpions) moving in different substrates (dry leaves, bark, sand, and desert soil) were found to create high bandwidth click-like sounds with maximum intensities of 60–85dB SPL at 100kHz at a distance of 10 cm [40, 41]. Because these studies were primarily focused on evaluating the intensity of prey-generated sounds they did not conduct spectral analysis or provide frequencies beyond 100 kHz and therefore we are unable to determine if suitable frequencies occur. A minimum frequency of 172 kHz is required for diffraction to begin occurring from a 1mm array. Both of these studies reported intensities at 100 kHz that may indicate usable frequencies of sufficient intensity could be present in prey-generated sounds. However, without testing we cannot be sure. Therefore we are not able to provide a practical comparison for the grooved versus groove-less gleaning bats that appear to rely completely on prey-generated sounds. We hypothesize that whether from prey-generated sources or from vocalizations, the large number of grooves found in most gleaning bats would affect the intensity consistent to the number of grooves involved. If suitable wavelengths are not present in either prey-generated sounds or their echolocation calls then the purpose of the grooves may have to be attributed to some other role like folding the ears.

In addition to echolocating bats, other bat species that do not echolocate (i.e. Pteropodidae) and species in other mammalian orders, have grooves in their ears (Table 4), often with much greater groove separation than those found in bat ears suggesting that their arrays affect lower frequencies than those typically used by echolocating bats. However, the grooves are not consistently represented within any given order or family, indicating that they may have evolved based on need and independent of phylogeny.

There are still many unanswered questions about how a groove array in the ear of a mammal influences acoustic signals and how that information may be of use, if at all. Basic wave theory leads us to believe the ear grooves are offering additional auditory capabilities over simple reflections, such as enhanced directionality and wavelength separation/selectivity. The different groove separation measurements may provide insight into the frequencies that are most useful to the species. Changing the angle of the array may help to monitor specific frequencies being diffracted. The highest groove numbers are found in echolocating bats known to glean prey from the ground or foliage. Based on our review of echolocating bats, the groove number appears to have a relationship to foraging strategy and therefore could help to clarify either guild definitions (in the case of bats) or provide greater insight into niche partitioning. Future studies evaluating the use of grooves in echolocating bats should try to document the presence of the frequencies that correspond to groove separations by ensuring the microphones are properly placed to overcome attenuation and capable of processing the information associated with those frequencies.

## Supporting information

S1 Table List of photos sourced from internet. (XLSX)
